# PROTOCOL: Are tools that assess risk of violent radicalization fit for purpose? A systematic review

**DOI:** 10.1002/cl2.1279

**Published:** 2022-10-07

**Authors:** Ghayda Hassan, Sébastien Brouillette‐Alarie, Sarah Ousman, Pablo Madriaza, Wynnpaul Varela, Emmanuel Danis, Deniz Kilinc, David Pickup, Eugene Borokhovski, CPN‐PREV team

**Affiliations:** ^1^ Department of Psychology Université du Québec à Montréal (UQÀM) Montréal Quebec Canada; ^2^ Department of Education Concordia University Montreal Quebec Canada

## Abstract

This is the protocol for a Campbell systematic review. The main objective of this project is to gather, critically appraise, and synthesize evidence about the appropriateness and utility of tools used to assess the risk of violent radicalization.

## 1 BACKGROUND

### 1.1 The problem, condition, or issue

Assessment of the risk of engaging in or desisting from a violent radicalization trajectory has evolved quickly in the last 10 years. “Standing on the shoulders of giants” (Logan & Lloyd, 2019) of what has been achieved in psychology and criminology over the last 50 years, scholars from the field of preventing violent radicalization have tried to import key lessons from violence risk assessment and management while taking into account the idiosyncrasies of their particular field (e.g., Borum, 2015).

The advantages of having reliable and valid tools to anticipate and mitigate the risk of violent radicalization—whether the outcomes of interest be ideological entrenchment or violent acting outs—cannot be underestimated. Law enforcement and intelligence professionals must assess persons of concern before they become involved in planning or executing attacks, and the criminal justice system must determine when and under what circumstances inmates may be released (Borum, 2015). Similarly, mental health and psychosocial professionals are now often called to perform radicalization risk assessments in the same way they would for suicidal or homicidal risks among their clients. While risk assessment in the security field is often used to guide surveillance, disruption, and/or sentencing, in the mental health and psychosocial field, it is mainly used to guide prevention and rehabilitative efforts.

In the field of correctional psychology, rehabilitative approaches emphasize risk assessment's relevance for correctional intervention and service delivery or, in other words, risk reduction via treatment, capacity building, and social reinsertion (Brouillette‐Alarie & Lussier, 2018). Initially dominated by a “nothing works” mindset (Martinson, 1974), the fields of psychology and criminology funded research on the determinants of effective correctional programming, leading to the identification of the risk‐need‐responsivity^1^ principles and the development of interventions that are able to reduce the risk of recidivism among judicialized persons (Andrews & Bonta, 2010). Interventions not based on risk‐need‐responsivity principles are generally ineffective and can sometimes lead to iatrogenic effects (Andrews & Bonta, 2010). By contrast, interventions that respect all three principles can achieve effect sizes (*r* = 0.26; Andrews & Bonta, 2010) comparable to those of psychological and medical interventions (Marshall & McGuire, 2003). The risk‐need‐responsivity principles of effective correctional intervention are rooted in reliable and valid assessments of the risk posed by judicialized individuals, particularly the sources or causes of that risk (i.e., such individuals’ criminogenic needs). Even though the lessons of correctional psychology cannot be imported “as is” in the field of preventing violent radicalization, they nevertheless highlight the importance of effective risk assessment tools to help structure prevention and intervention efforts.

Unfortunately, as of now, there are no gold standards in the risk assessment of violent radicalization. Multiple authorities in the field are critical of the viability of risk assessment, especially when it is of the actuarial type (Borum, 2015; Monahan, 2012). Five obstacles are commonly mentioned: (1) empirical research on risk and protective factors of violent radicalization is not sufficiently developed to provide a sound empirical basis for what to include and not include in tools; (2) research on risk factors comes from commonalities between individuals who have committed terrorist attacks, but appropriate validation would require that these characteristics be relatively absent of control groups that have not committed such attacks; (3) the low base rate of recidivism among violent radical offenders complicates predictive validity analyses for risk factors and tools; (4) violent radicalization trajectories can lead to multiple outcomes (i.e., radicalization of ideas, joining and participating in violent radical group activities, committing acts of violence, etc.) and the same predictors might not apply to the same outcomes; and (5) risk of violent radicalization might not be cumulative, in contrast to risk of general violence or criminal recidivism (Borum, 2015; Conley, 2019; Monahan, 2012; RTI International, 2018; van der Heide et al., 2019). To overcome these limitations, scholars have advocated for the development, validation, and use of structured professional judgment protocols over actuarial scales to assess the risk of violent radicalization (Borum, 2015; Monahan, 2012). At present, all tools that are designed to assess the risk of violent radicalization and that are used operationally by practitioners consist of structured professional judgment protocols (Scarcella et al., 2016): the Extremism Risk Guidance Factors (ERG 22+; National Offender Management Service, 2011); the Identifying Vulnerable People Guidance (IVPG; Egan et al., 2016); the Multi‐Level Guidelines (MLG; Cook et al., 2013, 2015); the Terrorist Radicalization Assessment Protocol (TRAP‐18; Meloy, 2017); and the Violent Extremism Risk Assessment (VERA‐2R; Pressman, 2009; Pressman et al., 2016; Pressman & Flockton, 2010).

Even though these tools rely on sound rationales, their level of empirical validation is currently lackluster—at least according to a systematic review conducted in 2016 by Scarcella and colleagues who found no validation studies which contained predictive validity analyses, arguably the most important criterion for risk assessment tools. Even though some validation studies used discriminant validity analyses (e.g., Meloy & Gill, 2016), convenience samples and retrospective coding of publicly available information were used rather than prospective studies with “real” participants. According to Scarcella et al. (2016), some widely used tools such as the VERA‐2R have no validation beyond face/content validity and inter‐rater agreement.

Research in the field of violent radicalization is evolving quickly, making assumptions that were considered “true” 5–10 years ago not so clear‐cut today. For example, most of the authors critical of violent radicalization risk assessment cited the lack of empirical research on risk and protective factors and the differential predictive validity of factors depending on the outcome of interest. Of note, a recent meta‐analysis of risk and protective factors of violent radicalization (Wolfowicz et al., 2020) included aggregated effect sizes separated by the following outcomes: radical attitudes, intention to act, and violent radical behaviors (e.g., attacks). Results indicated that not only did similar risk and protective factors predict both attitudes and behaviors, but also that socio‐demographic characteristics had less explanatory power than psychological‐ and personality traits‐related factors commonly found in violence risk assessment, thus contradicting many assumptions held in the field, namely, that risk factors for general violence alone may be insufficient or irrelevant in cases of violent radicalization. Similarly, the (unsubstantiated) assumption that risk of violent radicalization is not cumulative has been challenged by studies that found that risk and protective factors had incremental validity in the prediction of violent radical attacks toward persons (Jensen & LaFree, 2016). Hence, risk tools for violent radicalization that were created 10 years ago might rely on evidence that is being challenged in 2020, as data increasingly accumulates on types of violent radicalization beyond jihadist radicalization. Similarly, the level of validation of some risk tools, as reviewed by Scarcella et al. (2016), might have significantly evolved since these authors published their systematic review. This rapid evolution of scientific knowledge of tools to assess and monitor factors relevant to risk of violent radicalization warrants the production of a new systematic review, which will explore whether currently available tools are fit for purpose depending on the context, type of case assessed, and practitioner conducting the assessment.

### 1.2 Why it is important to do the review

The current systematic review will be of major relevance for practitioners receptive to the idea of using risk assessment tools but unsure of which to choose and for which context. Recent surveys of practitioners working in preventing violent radicalization report that most were open to using tools but uncertain if the available ones were adapted to their sector, setting, or clients (Hassan et al., 2020; Madriaza et al., 2018). Furthermore, the lack of validation of most tools as well as their significant monetary costs led some teams to develop homemade scales or rely purely on professional judgments. Knowing the potential pitfalls of assessing risk using unstructured clinical judgment (Dawes et al., 1989; Grove et al., 2000), it would be wise to answer practitioners’ questions concerning violent radicalization risk tools. Our systematic review could also enable assessors to avoid the potential iatrogenic effects associated with using tools that are not fit for purpose for certain populations or contexts.

The costs associated with misevaluating risk are numerous. Risk overestimation can lead to more surveillance, stigmatization, unjustified repressive practices, longer than necessary sentences, and waste of funds on interventions that are not only unnecessary but also potentially harmful (Andrews & Bonta, 2010; Brouillette‐Alarie & Lussier, 2018). Risk underestimation, in turn, can result in premature releases and new victims (Gendreau et al., 1996; Hanson, 2009). Even though it is unrealistic to assume that each recidivism case could have been prevented with better assessment or decision‐making, it is important for clinicians, practitioners, and evaluators to be able to attest that their decisions were based on empirically validated procedures and high ethical standards in risk assessment.

Potential risk overestimation is especially important in the context of violent radicalization because base rates are so low compared to other types of outcomes such as criminal recidivism (Borum, 2015). This makes prediction especially challenging, as statistical models usually underperform when base rates are very low. Therefore, an investigation of the potential false positive of violent radicalization risk tools is paramount.

In sum, the current systematic review will have implications for practitioners, decision‐makers, public safety, and even potential clients in the field of violent radicalization. Evidence permitting, it will advise practitioners and deciders concerning which tools to use, which tools to avoid, and in which context. It will also potentially ease clinicians’ concerns regarding risk tools or, conversely, raise their vigilance toward tools that are problematic. In both cases, the endeavor should contribute to better assessments of risks in the field of violent radicalization.

### 1.3 How this review might inform or supplement what is already known in this area

Searches for existing relevant systematic reviews and meta‐analyses were made in Google using the following search strings: “risk assessment violent radicalization systematic review” and “risk assessment violent radicalization meta‐analysis.” Results of the first five pages for each string were reviewed by the research team. Google was preferred to Google Scholar to ensure adequate coverage of the gray literature.

Five systematic reviews (Desmarais et al., 2017; Gill et al., 2021; Lösel et al., 2018; Misiak et al., 2019; Vergani et al., 2020) and two meta‐analyses (Emmelkamp et al., 2020; Wolfowicz et al., 2020) on risk and protective factors for violent radicalization were found. Although these systematic reviews and meta‐analyses are of relevance for examining the face and content validity of risk tools, they are beyond the scope of our systematic review, which focuses on tools rather than individual factors.

Only one systematic review on tools that assess the risk of violent radicalization was found: Scarcella et al. (2016). The review explored the level of psychometric validation of instruments that identify risk factors of terrorism, extremism, radicalization, authoritarianism, and fundamentalism. The authors found four instruments that specifically assess violent radicalization risk (e.g., VERA‐2R) and 17 research measures/instruments that assess outcomes related to violent radicalization (e.g., Right‐Wing Authoritarianism Scale [RWA]; Altemeyer & Hunsberger, 1992). As mentioned earlier, Scarcella et al. (2016) concluded that the empirical validation of most scales, especially those designed specifically to assess the risk of violent radicalization, was in its infancy. Even though they concluded that more validation was needed, they did not provide recommendations for practitioners who could be looking to use violent radicalization risk assessment tools. Moreover, some risk tools (e.g., MLG) were omitted while others may have been published since then. Considering the incredibly fast pace with which the field is evolving (i.e., many studies on violent radicalization risk tools have been published in the last 5 years), proposing recommendations based on evidence collected in 2016 would be, at the very least, out of date, and, at most, potentially irresponsible. Finally, Scarcella et al. (2016) only briefly discussed criterion‐validity findings (e.g., predictive, concurrent, and convergent validity)—arguably the most important validation criterion for professionals looking for guidelines on which tools to use and with whom.

Although it is neither a systematic review nor meta‐analysis, the contribution of Logan and Lloyd (2019) must also be noted. In their paper, the authors first contextualize the tasks of assessing, understanding, and managing risk in the field of violent radicalization based on the progress made in the fields of correctional psychology and criminology. Then, they map the risk assessment tools used by practitioners in the field of violent radicalization, describe their intended use, and relate studies attesting to their validation. Finally, they suggest guidance on how to ethically conduct risk assessment with individuals on violent radicalization trajectories, appraise the quality of existing evidence concerning the validation of available risk tools, and plan future evaluations of risk tools in the field. However, as most summaries of violent radicalization risk tools (e.g., Conley, 2019; RTI International, 2018; van der Heide et al., 2019), the methods used to search for relevant literature or collect and analyze data were not systematic. Therefore, the current systematic review aims to improve on their important work by structuring data collection and analysis, as well as ensuring that the literature search will be up to date.

## 2 OBJECTIVES

The main objective of this project is to gather, critically appraise, and synthesize evidence about the appropriateness and utility of tools used to assess the risk of violent radicalization. The specific questions of our systematic review are as follows:

1. What are the tools used to assess the risk of violent radicalization? These will include:
  a. Tools specifically developed to assess the risk of violent radicalization.
  b. Tools *not* specifically developed for that purpose (e.g., from other fields), but for which studies have tested the link with violent radicalization outcomes.
2. What is the reliability of these tools?
3. What is the validity of these tools?
4. Based on the psychometric properties of tools, as validated with specific populations and in specific contexts, are they fit for purpose for such populations and contexts? In other words, what are the advantages and disadvantages associated with the use of these tools for public safety, practitioners, and individuals on a trajectory toward violent radicalization?

The logic model underlying the objectives of the current review can be found below. Each component of the logic model is described in detail in the Methodology section.


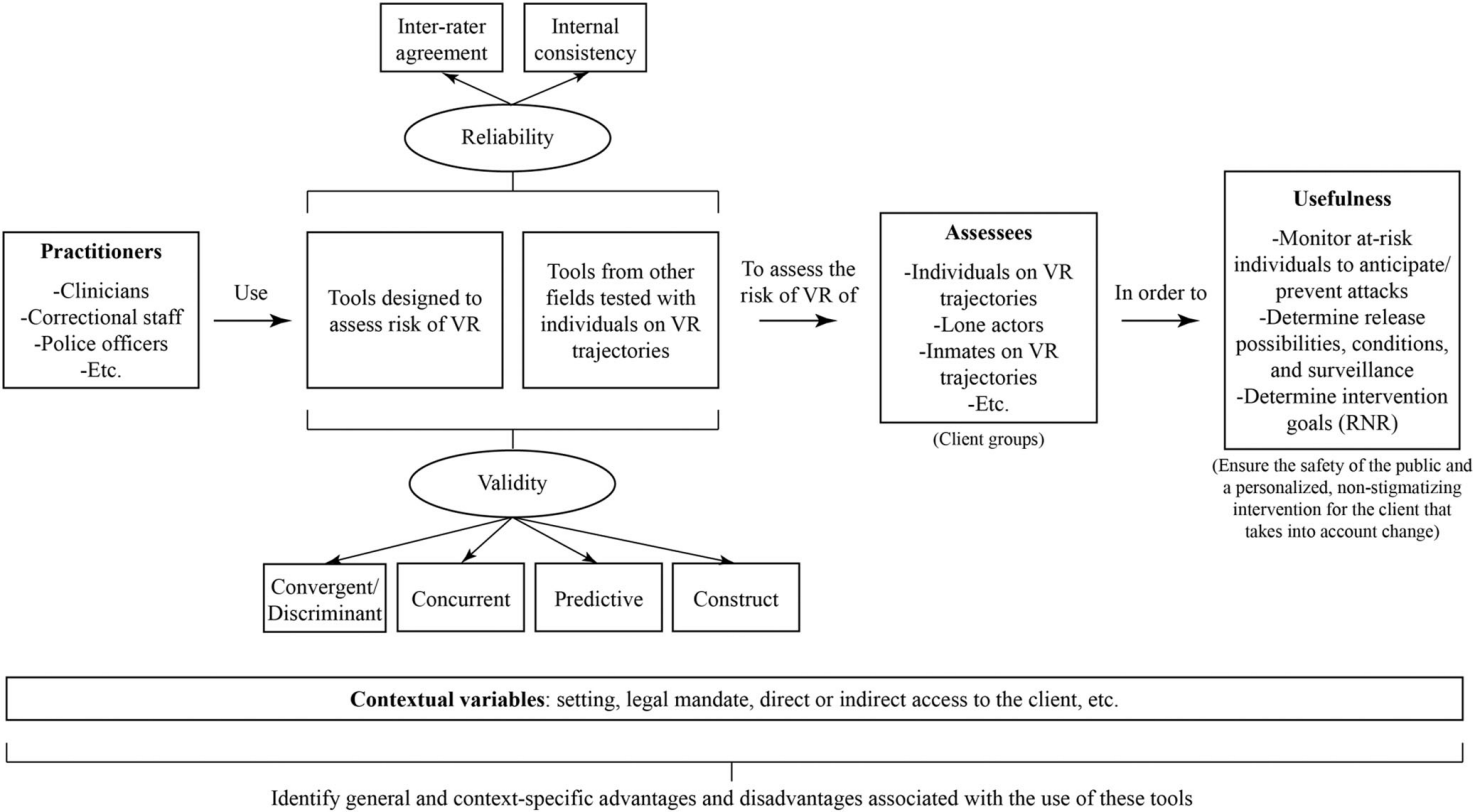


## 3 METHODOLOGY

### 3.1 Phenomena of interest

The phenomena of interest to the current systematic review are risk assessment tools designed to assess the risk of violent radicalization. We are also interested in studies that verified if risk assessment tools from other fields, such as psychology and criminology, could be applicable and useful in the risk assessment of individuals on a violent radicalization trajectory. In the section below, we propose definitions of violent radicalization, risk assessment, risk/protective factors, and risk tools—whether actuarial or using structured professional judgment.

Definitions for fundamental risk assessment notions are based on the following sources: Andrews and Bonta (2010), Brouillette‐Alarie and Lussier (2018), Douglas and Skeem (2005), Gendreau et al. (1996), Hanson (2009), and Hanson and Harris (2000).

#### 3.1.1 Violent radicalization

Non‐linear process by which an individual or a group (including a state) undergoes systemic transformations (e.g., behavioral, socio‐economical, psychological, identity‐based, political, and/or ideological) that lead them to support or facilitate the use of violence toward an individual or a group to further their cause and bring about individual or societal changes (Hassan et al., 2021). In empirical studies, violent radicalization is often operationalized as violent radical attitudes, intent to act, and violent radical behaviors (Wolfowicz et al., 2020). Preliminary screening of studies revealed that outcomes included planning versus executing attacks (Meloy & Gill, 2016), protesting peacefully versus violently (Jensen & LaFree, 2016), and harboring violent radical attitudes or not (Altemeyer & Hunsberger, 1992). Even though the initial screening stage did not find staying versus leaving one's extremist group and criminal recidivism as outcomes, we anticipate they are likely to be encountered.

#### 3.1.2 Risk assessment

Estimating the likelihood of an undesirable future event with indicators from the individual's past or present situation. Risk assessment reduces uncertainty about the future by aggregating sources of information that are predictive of a specific type of event (in this case, violent radical attitudes and behaviors). In social science, risk assessment is inherently probabilistic: It does not predict the future but compares the risk of an event happening depending on the presence or absence of certain variables.

#### 3.1.3 Risk factors

Past or present characteristics of individuals that increase their risk of engagement or entrenchment in violent radicalization trajectories.

1. *Static risk factors*: Features of individuals’ histories that are predictive of engagement/entrenchment in violent radicalization trajectories but not amenable to change.
2. *Dynamic risk factors*: Dynamic risk factors (or criminogenic needs) are aspects of a person or its social environment that, when modified, can result in changes in engagement/entrenchment in violent radicalization trajectories. Because violent radicalization is a non‐linear process, when it comes to assessment, dynamic (temporally sensitive) risk factors should be preferred to static ones. There are two types of dynamic risk factors: stable and acute risk factors.
  a. *Stable risk factors* tend to endure for months or years and require significant efforts to be modified.
  b. *Acute risk factors* can change over weeks, days, or even hours and signal the timing of heightened risk states. These factors are not necessarily related to long‐term risk; they are temporary disruptions in the individual's psychological state or social environment that can precipitate relapse.

#### 3.1.4 Protective factors

Characteristics of individuals and their social environment that are associated with reduced chances of engagement/entrenchment in violent radicalization trajectories or that can be protective against risk.

#### 3.1.5 Risk tools

Ways of identifying, combining, and weighting risk and protective factors to assess the risk of engagement/entrenchment in violent radicalization trajectories. Importantly, to be eligible for this systematic review, risk tools had to be useable by practitioners to assess cases involving individuals. In other words, tools designed to assess the risk of a building being targeted, for example, were ineligible, with the same being true for tools operating at a group level (e.g., risk of two groups clashing) or requiring access to large databases to function (e.g., tools that scour thousands of Twitter posts from multiple users). Scales developed for research purposes only (i.e., usually put together by recoding or summing already existing variables in a data set for theory testing, and on which no scoring sheet or coding manual exists) were ineligible for inclusion, the same being true for self‐revealed psychometric scales that assess outcomes related to violent radicalization, such as the RWA or the Activism and Radicalism Intention Scale (ARIS; Moskalenko & McCauley, 2009). By contrast, the ERG 22+, IVPG, MLG, TRAP‐18, and VERA‐2R are prime examples of eligible risk tools. Finally, if studies verified whether existing risk tools from the fields of psychology and criminology were applicable to individuals on violent radicalization trajectories, they will be included. However, other studies utilizing these tools (i.e., with non‐radicalized samples) were rejected.

#### 3.1.6 Actuarial assessment

Actuarial tools aim to reliably determine the level of risk by mechanically combining empirically validated predictors. This method is considered atheoretical because an item's main inclusion criterion in a scale is its statistical association with the outcome of interest and not its theoretical relevance. Each item is weighted in advance by the developers. Coding rules are very specific, leaving little room for professional input. The final risk score is equal to the sum of the items presented by the assessee. Once the total score has been computed, the evaluator can check the instrument's normative tables so as to clearly communicate their client's risk level to deciders. As mentioned in the Background section, actuarial assessment has been largely overlooked or discarded by scholars in the violent radicalization field.

#### 3.1.7 Structured professional judgment

Structured professional judgment tools provide a framework for clinical judgment by specifying which risk and protective factors to assess and how. Therefore, this method solves one of the main issues of unstructured clinical judgment: the use of irrelevant factors and the omission of potentially important ones. Factors included in structured professional judgment tools must be empirically validated and offer legitimate avenues for intervention. Contrarily to actuarial scales, structured professional judgment tools do not have mechanical compilation procedures. Rather than summing factors whose relative weight is specified in advance, the evaluator is required to make a holistic judgment about the risk of the assessee based on the risk and protective factors present. There is no normative table that links risk scores to anticipated recidivism rates. As of now, all tools used operationally by professionals to assess violent radicalization risk are structured professional judgment protocols (Scarcella et al., 2016).

### 3.2 Criteria for considering studies in this review

Note that the same search strategy and inclusion/exclusion criteria will be used for all four of this systematic review's objectives.

**Table:** 

Included	Excluded
Risk tools for violent radicalization Risk tools from other fields tested with violent radical participants	Risk tools from other fields tested with participants not on a violent radicalization trajectory
Tools operationally usable by clinicians for cases involving individuals	Research scales not operationally usable by clinicians Tools for risk that do not involve individuals (e.g., risk of a building being targeted) Tools requiring large social media databases Self‐revealed psychometric scales
Primary data, including indirect primary data (e.g., triangulation of publicly available data)	Secondary data (i.e., literature reviews, systematic reviews, meta‐analyses—references will be checked, however)
Quantitative studies	Qualitative studies Nonempirical papers (e.g., theoretical or opinion pieces)
Comprises data on the types of reliability and validity eligible in this systematic review (see the Outcomes section)	Does not comprise data on eligible types of reliability and validity (see the Outcomes section)

We will include studies with primary data resulting from the quantitative examination of the reliability and validity of tools used in a violent radicalization context. We will also include evaluations of tools used for general violence if tested with participants on a violent radical trajectory. We initially planned to include qualitative research designs, but a survey of initial search results indicated that very few or none were available in the context of risk tool validation. Furthermore, only tools useable by practitioners aiming to assess cases of individuals will be eligible. This means that the following will be ineligible: (a) tools designed to assess the risk of a building being targeted; (b) tools that operate at a group level; (c) tools that require access to large databases to function; (d) scales developed for theory validation only; and (e) self‐revealed psychometric scales assessing constructs related to violent radicalization.

Beyond limiting ourselves to studies comprising primary quantitative data (including the quantitative sections of mixed‐method studies) about the reliability and validity of eligible risk tools, we will not impose any restrictions on study design, type, method, or date (up to December 31, 2021) because the state of the literature is such that doing so could lead to the inclusion of only a small number of studies that do not give a clear picture of what is being done in the field. Eligible studies will be categorized into the following groups (in decreasing order of methodological robustness):

1. Prospective data on reliability and validity using samples recruited in clinical and/or prison settings (probably very rare).
2. Retrospective data on reliability and validity using samples recruited in clinical and/or prison settings (probably rare).
3. Retrospective data on reliability and validity using samples built by compiling publicly available information about terrorists/violently radicalized individuals (e.g., already existing terrorist databases or datasets put together by compiling journal articles about terrorist cases; probably common).

We will exclude manuscripts where the authors reflect on a tool, as such reflections do not comprise primary empirical data. We will, nevertheless, take note of the authors’ main conclusions, should they prove relevant for our discussion section. The same will be done for qualitative studies.

To reduce “publication bias” (Tanguy et al., 2014), our review will include not only articles published in peer‐reviewed journals but also gray literature found by searching the Web using Google for studies and reports from government and non‐government organizations. Our review will exclude systematic and literature reviews, as these constitute secondary data. We will, however, scour the references of such reviews to ensure that our search strategy found all the relevant studies.

### 3.3 Population and context

To be eligible for this review, studies on assessment tools will need to comprise individuals involved in or at risk of becoming involved in violent radicalization trajectories, whether or not those tools were specifically developed to assess violent radicalization risk. If a study only comprises practitioners or stakeholders rather than clients (e.g., a study asking evaluators about their experience using a tool), it will be eligible on the condition that the tool was designed to assess the risk of violent radicalization. Other than that, no other exclusion criteria concerning participants and contexts will be applied. When full‐text coding, we will take detailed notes on participants’ characteristics—be they clients or practitioners—and contextual variables, wherever feasible, to thematically aggregate evidence.

#### 3.3.1 Client groups

There are several characteristics that may affect the appropriateness and/or usefulness of violent radicalization risk tools with certain client groups. For example, the TRAP‐18 was developed to assess the risk of lone‐actor terrorists and, thus, may not be effective for group‐based acting out (Meloy & Gill, 2016). Similarly, some tools may be more effective with inmates and offenders released in the community than with individuals who have not yet started their criminal careers. Some may be more effective with certain types of violent radicalization than others (e.g., far right, far left, religiously based, nationalist, single issue, etc.). Recommendations for practitioners will consider which tools are appropriate for which client groups, depending on the level of psychometric validation.

#### 3.3.2 Practitioners

As with client groups, violent radicalization risk tools are likely to be more relevant for (or limited to) certain professionals and sectors. For example, in general violence risk assessment, the Psychopathy Checklist—Revised (Hare, 2003) or START (Webster et al., 2006) are exclusive to psychologists and psychiatrists.

#### 3.3.3 Other contextual variables

In addition to client group and practitioner type, other factors could influence the effectiveness of violent radicalization risk tools. These factors include the setting (e.g., mental health institution vs. correctional vs. in the community), amount of data to which the assessor has access (including if the tool can be scored or not without an interview), and the legal and/or professional mandate of the assessor, and so on. If data about contextual variables is available, it will be taken into consideration in the recommendations to emerge from this review.

### 3.4 Outcomes

In this review, the main outcome of interest will be the level of empirical validation of each tool. Empirical validation will be divided into the following dimensions: reliability (inter‐rater agreement, internal consistency) and validity (convergent, discriminant, predictive, concurrent, construct).

#### 3.4.1 Reliability

Degree to which the measure of a construct is consistent or dependable.

1. *Inter‐rater agreement*: Measure of consistency between two or more independent raters (e.g., assessor) of the same construct.
2. *Internal consistency*: Measure of consistency between different items of the same construct.

#### 3.4.2 Validity

Extent to which a measure adequately represents the underlying construct it is supposed to measure.

1. *Convergent*: Closeness with which a measure relates to (or converges on) the construct it is purported to measure.
2. *Discriminant*: Degree to which a measure does not measure (or discriminates from) other constructs it is not supposed to measure.
3. *Predictive*: Degree to which a measure successfully predicts a future outcome it is theoretically expected to predict.
4. *Concurrent*: How well one measure relates to another concrete criterion that is presumed to occur simultaneously.
5. *Construct*: Construct validity can be construed as an overarching type of validity that is defined by the extent to which scores on the instrument are indicative of the theoretical construct. However, in the context of this systematic review, construct validity will mainly encompass examinations of the latent structure of a tool as obtained by, for example, factor analyses.

We will document the types of practitioners, clients, and contexts on which the validity of the tools has been assessed.

### 3.5 Search methods for identification of studies

In consultation with a library science expert, we developed a search strategy aimed to target an array of bibliographic databases and gray literature resources. Wherever possible, we will use controlled vocabulary terms from database thesauri and adapt the strategy by database to make full use of its features.

#### 3.5.1 Electronic searches

We will conduct searches in a variety of bibliographic databases, both subject‐specific databases and general multidisciplinary databases. The proposed list is as follows: Academic Search Complete (EBSCO), Criminal Justice Abstracts (EBSCO), Education Source (EBSCO), ERIC (EBSCO), International Security & Terrorism Reference Centre (EBSCO), Medline (EBSCO), Violence and Abuse Abstracts (EBSCO), National Criminal Justice Reference Service (NCJRS; https://www.ojp.gov/ncjrs/virtual-library/search), ProQuest Central, ProQuest Dissertations and Theses Global, PsycINFO (Ovid), Sociological Abstracts (ProQuest), CINCH Australian Criminology Database (Informit), and the Web of Science platform's core collection (SCI‐EXPANDED, SSCI, A&HCI, CPCI‐S, CPCI‐SSH, and ESCI). While the searches will employ standard Boolean logic, they will be tailored to the features of each database, making use of available controlled vocabulary and employing proximity operators where possible.

Our search strategy is divided into three blocks. The first block is a proximity search for terms that represent “risk” and “assessment,” which need to be maximum three words apart. The second block is the “radicalization” and accompanying synonyms block. The third block comprises the names of risk tools relevant to the field of violent radicalization that were found in preliminary searches. To be eligible in our systematic review, a manuscript needs to satisfy (BLOCK 1 AND BLOCK 2) OR BLOCK 3.

Searches will be conducted in English, but no languages will be excluded from the results (i.e., if a Spanish paper contains an English abstract that is identified by our English search string, it will be included). The search fields will include title, abstract, keywords, and subject/indexing. Endnote will be used to index the results.

The following example is the search run planned for the PsycINFO (Ovid) database:

**BLOCK 1**

((assess* or predict* or evaluat* or screen* or tool* or protocol* or scale* or instrument* or calibrat* or index* or measur*) **adj3** (risk* or vulnerabil* or recidiv* or dangerous* or threat* or need*)).ab,hw,id,mh,ot,ti.

**BLOCK 2**

(radicali* or extremis* or fundamentalis* or terroris* or “hate crime*“ or “religious violence*“ or “political violence*“ or “ideological violence*“ or “environmental violence*“ or “racist violence*“ or “separatist violence*“ or “far right” or “right wing” or “alt right” or “radical right” or “extreme right” or “white supremac*“ or “neo nazi*“ or neonazi* or “anti semiti*“ or antisemiti* or “anti‐semiti*“ or “left wing” or “far left” or “alt left” or “anti fa” or antifa* or anarch* or “anti capitalis*“ or anticapitalis* or jihadis* or islamis* or salafis* or “lone wolf*“ or “lone actor*“ or “lone offend*“ or “suicide bomb*“ or “suicide attack*“ or “mass shoot*“ or indoctrinat* or “foreign fight*“ or martyr*).ab,hw,id,mh,ot,ti.

**BLOCK 1 AND BLOCK 2**

**BLOCK 3**

(“Activism and Radicalism Intention Scales” or “Building Resilience to Violent Extremism” or “BRAVE 14” or BRAVE14 or “Extremism Monitoring Instrument” or “EMI 20” or EMI20 or “Extremism Risk Guidance” or “Extremism Risk Guidelines” or “ERG 22+“ or ERG22+or “IAT 8” or IAT8 or “Identifying Vulnerable People Guidance” or “Intratextual Fundamentalism Scale” or “IR 46” or IR46 or “Militant Extremist Mindset” or “Multi level Guidelines” or “Multi‐Dimensional Fundamentalism Inventory” or “Radicalisation Risk Assessment in Prisons” or “Référentiel des indicateurs de basculement dans la radicalisation” or “Religious Fundamentalism Scale” or “Significance Quest Assessment Tool” or “Significance Quest Assessment Test” or SyFoR or “Terrorist Radicalization Assessment Protocol” or “TRAP 18” or TRAP18 or “Violence Threat Risk Assessment” or “Violent Extremism Beliefs Scale” or “Violent Extremism Risk Assessment” or “VERA 2” or VERA2* or “Vulnerability Assessment Framework”).ab,hw,id,mh,ot,ti.

**(BLOCK 1 AND BLOCK 2) OR BLOCK 3**

limit 5 to yr = “1860 ‐ 2021”

#### 3.5.2 Searching other resources

The gray literature search strategy will be divided into four parts. Part 1 will consist of inputting the following search string in Google: “risk assess AND (radicalization OR radicalisation OR extremism) filetype:pdf.” Google will be preferred to Google Scholar to ensure better coverage of manuscripts not published in indexed scientific journals. Search results will be scoured until five pages of irrelevant results come out (with 10 results per page). Part 2 will be looking for relevant articles in the Violent Extremism Evaluation Measurement framework, available at https://www.rand.org/randeurope/research/projects/violent-extremism-evaluation-measurement-framework-veem.html. Part 3 will involve searching webpages and research repositories relevant to violent radicalization. The list of webpages/repositories is based on another Campbell Collaboration systematic review led by members of the same team as this one. The list goes as follows: Alliance for Peacebuilding – Monitoring and Evaluation of CVE; American Bar Association Rule of Law Initiative (ABA ROLI); Brennan Center for Justice; Center for Evidence Based Crime Policy (CEBCP); Center for Strategic and International Studies; Center on Global Counterterrorism Cooperation (CGCC); Center on International Cooperation at New York University; Comité interministériel de prévention de la délinquance et de la radicalisation (CIPDR); Cleen Foundation (Nigeria); COWI; Design Monitoring and Evaluation for Peacebuilding; French Ministry of Interior Publications Database; Geneva Centre for Security Policy (GCSP); German National Center for Crime Prevention; Global Center on Cooperative Security (GCCS); Global Counter Terrorism Forum (GCTF) Tools; Global Counter Terrorism Forum Violent Extremism (Hedayah); https://defence.gov.au/; https://educateagainsthate.com/UK; https://en.unesco.org/preventing-violent-extremism; https://georgetownsecuritystudiesreview.org/; https://www.coe.int/en/web/counter-terrorism; https://www.crisisgroup.org/who-we-are; https://www.csis.org/analysis/counterterrorism-measures-and-civil-society; https://www.dhs.gov/; RUSI; https://www.start.umd.edu/; Institute for strategic dialogue (ISD); International Centre for the Study of Radicalisation (ICSR); International Centre of Excellence for Countering Violent Extremism; Institute for security studies ISS South Africa; Ministry of Foreign Affairs of Denmark; Mounted Police (RCMP); Organization for Security and Co‐operation in Europe (OSCE); Peace and Stabilisation Fund (Denmark); RAND; Search for Common Ground; The Campbell Collaboration; UK Home Office Research Database; UK Ministry of Defence; Union Européenne (https://op.europa.eu/fr/more-search-options); United Nations Development Programme (UNDP); United Nations Office for Drugs and Crime's Terrorism Prevention Branch (UNODC); USAID; Violence Prevention Network (Germany); World Organization for Resource Development and Education (WORDE). Part 4 will involve conducting Google searches for each violent radicalization risk assessment tool known by the research team. Here is an example of a search string for the ERG22+: “extremism risk guidance filetype:pdf.” Finally, part 5 will comprise thoroughly searching the reference sections of literature and systematic reviews of risk assessment tools in the field of violent radicalization, as well as the references of all included studies in this systematic review.

Finally, hand searches of relevant papers will be conducted in the Journal for Deradicalization (https://journals.sfu.ca/jd/index.php/jd/index), an open source and well‐regarded journal in the field that is, however, not indexed in bibliographic databases.

### 3.6 Data collection and analysis

#### 3.6.1 Description of methods used in primary research

To illustrate how our inclusion/exclusion criteria function and how the results of a manuscript might be classified into our outcomes category, the Powis et al. (2021) paper entitled “An examination of the structural properties of the Extremism Risk Guidelines (ERG22+): A structured formulation tool for extremist offenders” will be reviewed. The paper explores the construct validity and internal consistency of the ERG22+ in a sample of 171 individuals convicted of an Islamic extremist‐related offense in the UK. Face‐to‐face interviews with convicted individuals were conducted, and official records were read by researchers. Exploratory factor analysis, multidimensional scaling, and Cronbach's alpha analyses were done.

The article was deemed eligible as it was (a) about a risk tool for violent radicalization (ERG22+); (b) the tool was usable by clinicians for cases of individuals potentially on a trajectory toward violent radicalization; (c) the article comprised primary data; (d) was quantitative; and (e) contained data on eligible types of reliability and validity for the ERG22+.

The article contained two types of outcomes relevant to our systematic review: data on internal consistency (reliability) and data on construct validity (factor analysis and multidimensional scaling). Internal consistency was good (α = 0.80), and exploratory factor analysis and multidimensional scaling suggested the presence of multiple (five to seven) dimensions in the scale. The fit of the multidimensional scaling solution was slightly below the threshold mentioned in the Methods section. The article concluded that the current three‐factor conceptual division of the scale was not found in empirical divisions of the scale.

As to conclusions that might emerge from our review of this article, we anticipate the following: (a) internal consistency of the scale was good in a sample of UK‐based individuals involved in violent Islamist radicalization; and (b) the three ERG22+ subscales/domains proposed by the instrument's creators were not found in empirical divisions of the scale. Thus, this type of construct validity was not clearly established.

It is important to note that to recommend a scale with a specific population (e.g., individuals involved in violent Islamist radicalization), data on more types of reliability and validity—namely, inter‐rater reliability and predictive validity—would need to be found in other studies.

#### 3.6.2 Selection of studies

Selecting admissible studies will be performed by three research assistants who will independently screen the abstracts of all manuscripts identified in the literature search. Each manuscript will be rated as “no,” “maybe,” or “yes” according to a screening tool based on our inclusion/exclusion criteria (see below). Disagreements (i.e., one research assistant having a different response than the others) will be dealt with in a meeting with the co‐lead researcher until consensus is reached.

**Table:** 

**Study Inclusion Screening Tool**	
Is the study about a risk tool for violent radicalization or a risk tool from another field that was tested with violent radical participants?	No/Maybe/Yes
Is that tool operationally usable by a clinician wishing to assess the risk of a violently radicalized individual?	No/Maybe/Yes
Does the study comprise quantitative data about the reliability and validity of this tool?	No/Maybe/Yes
**If a “no” is present, exclude**.	
**If a mix of “yes” and “maybe,” maybe include**.	
**If all “yes,” include**.	
Is the study based on primary data (including indirect primary data such as triangulation of publicly available data)?	Unknown/Primary/Secondary
**If secondary, exclude but check references**.	

Training will be provided to research assistants before the start of the selection process. After 20% of articles have been coded, the “initial” inter‐rater agreement will be computed using Fleiss's (1971) *κ*. Another round of training will be provided to research assistants at that stage, and the “final” inter‐rater agreement on another set of articles (20%) will then be computed. Both will be reported in the systematic review.

All studies rated as “maybe” or “yes” after the disagreements have been resolved will be selected for full‐text screening. During the full‐text screening, assistants will confirm that the studies meet all the inclusion/exclusion criteria and comprise data relevant to our outcomes of interest—something which is often not easy at the initial screening stage. If all this is confirmed, the selected studies will be coded in full. Each full‐text study that a research assistant selected for exclusion will also be screened by the co‐lead researcher to ensure that no relevant data is left out of the systematic review.

Lastly, the PRISMA (http://www.prisma-statement.org) template will be used to record the results of the literature searches in a flowchart.

#### 3.6.3 Data extraction and management

Each retained study will be processed in an Excel coding sheet where the following information will be extracted:

(1) Document ID, title, authors, year of publication, and place published.
(2) Relevant risk tools studied in the paper.
(3) Data source (meetings with participants, private institutional records, triangulation of publicly available data).
(4) Study design (cross‐sectional vs. longitudinal, retrospective vs. prospective).
(5) Sample characteristics (*N*, gender, age, country, ethno‐racial group, education, employment status, religious/ideological affiliation, other relevant information).
(6) Outcomes.
  a. Reliability (inter‐rater agreement, internal consistency).
  b. Validity (convergent, discriminant, concurrent, predictive, construct).
(7) Recommendations of authors (concerning the tool, for practitioners, for future research, other).
(8) Limitations mentioned by authors.
(9) Coder ID and coding date.

Each of these categories of information will correspond to one column in the coding sheet, with studies being entered on rows. If a study assesses the psychometric properties of multiple eligible tools, it will be listed in multiple rows (once per tool). Because some psychometric properties cannot be simply summarized in one clear numerical variable (e.g., the results of a factor analysis), reliability and validity data will initially be entered as text with clear references to the appropriate tables for situations where aspects of the data cannot be summarized in text (e.g., a factor loading table).

If the need to meta‐analyze reliability and validity coefficients arises (see the Data Synthesis section), we will build a data set in SPSS that will comprise the relevant numerical coefficients based on the text‐format information present in the Excel coding sheets. It will then be imported in the software used for meta‐analysis (see the Data Synthesis section).

Only studies written in languages spoken by at least one member of our research team will be processed in the coding sheets, as relying on automatic translation services such as Google Translate or DeepL would be unlikely to be sufficiently precise for detailed scientific scrutiny. We will, however, index and make a list for readers of the papers that would have been eligible otherwise.

#### 3.6.4 Assessing the methodological limitations of included studies

The methodological quality of studies, also known as “risk of bias,” will be assessed through a modified and shortened version of the COSMIN Risk of Bias checklist (Mokkink et al., 2018; Prinsen et al., 2018; Terwee et al., 2018). Because the COSMIN Risk of Bias checklist was created for the medical field, trying to use it “as is” in an emergent field such as that of violent radicalization would make the tool unfit for purpose (i.e., too many items would be rated N/A due to lack of information). Therefore, the co‐lead researcher, who has substantial expertise in psychometry and quantitative research, selected and adapted items from the COSMIN Risk of Bias checklist deemed relevant to the state of the literature in the field. The modified version of that tool can be found in Supporting Information: Appendix A.

The risk of bias checklist used in this systematic review will be scored by research assistants under the supervision of the co‐lead researcher. Training in psychometry and quantitative research will be provided to research assistants beforehand, if necessary. Research assistants will be encouraged to ask for help with any item that might prove difficult to score. When this happens, the co‐lead researcher will make sure to review the risk of bias checklist of the study in question.

We will not exclude studies based on their methodological robustness a priori because one of the main goals of this systematic review is to critically appraise and provide guidance on the level of validation of risk tools in the field of violent radicalization. If lower‐quality studies were to be excluded from this review, it would present a picture of the literature that is artificially optimistic. However, recommendations concerning the use of each tool will be informed by the quality of studies attesting to its psychometric validation.

We do not anticipate finding studies in which the authors of this systematic review are involved, but should that happen, such authors will not be involved in the risk of bias assessment of their study.

#### 3.6.5 Data synthesis

The data to be synthesized will be the reliability and validity of risk tools. We anticipate quantitative summaries to be applicable for inter‐rater agreement, internal consistency, convergent, discriminant, concurrent, and predictive validity. Convergent, discriminant, concurrent, and predictive validity might, however, prove problematic due to the heterogeneity of comparators/outcomes used. However, if possible and theoretically sound, these psychometric indices will be meta‐analyzed to extract the central tendencies concerning the psychometric validation of specific risk tools.

Meta‐analysis will be conducted when two or more studies report the same psychometric property for the same risk tool and in similar populations (i.e., samples compatible in relation to the type of violent radical ideology). Inter‐rater agreement will be meta‐analyzed according to Sun's (2011) guidelines for Cohen's κ, while internal consistency will be meta‐analyzed according to Bonett's (2010) guidelines for Cronbach's alpha. For convergent, discriminant, concurrent, and predictive validity, we anticipate that multiple effect size measures will be reported in articles. For studies that use ordinal/continuous variables to compare groups, Cohen's *d* or Hedge's *g* will be computed using means, standard deviations, and sample sizes (Borenstein et al., 2009). For studies using correlations, we will convert Pearson's *r* to Fisher's *z* using the formulas proposed by Lipsey and Wilson (2001). For areas under receiver operating characteristic curves, Zhou, Obuchowski, and McClish (2002) guidelines will be used. Effect sizes will be aggregated using the Comprehensive Meta‐Analysis (Borenstein et al., 2014) and MedCalc (MedCalc Software Ltd, 2021) software according to the random effects analytical model.

Then, heterogeneity will be investigated (see the section below) for contextual variables (e.g., setting, type of practitioner using the tool). If a study is found to be excessively unreliable (cf. risk of bias), it will be left out of the aggregation of findings. We will keep an eye out for redundant manuscripts, that is, studies of the same psychometric properties of the same tool with the same sample. If found, only the most robust studies will be kept in the meta‐analysis in order not to over‐represent a sample.

For situations where meta‐analysis cannot be conducted, effect sizes and confidence intervals will be reported and discussed, while acknowledging the limitations of the scope of the results. After data synthesis, we should be able to respond to the following question: “Is a risk tool reliable and valid enough to be recommended for use with a specific population in a specific context?” We will detail when risk tools are appropriate (validated), when they are not (validation was tried but failed), or when it is unknown (studies with said population/context were not yet conducted or evidence is too unreliable to infer meaningful conclusions).

#### 3.6.6 Assessment and investigation of heterogeneity

As already mentioned, we anticipate violent radicalization risk tools to display different psychometric properties depending on contextual variables (e.g., setting or type of practitioner). For such cases, heterogeneity will be taken into account during quantitative synthesis using *I* ^2^, *τ* ^2^, and *Q* statistics (Borenstein et al., 2009).

Even if we cannot conduct meta‐analysis due to the lack of appropriate data, we will consider populational and contextual variables in our recommendations, as they are key for day‐to‐day risk assessment practice.

#### 3.6.7 Assessment of certainty of the evidence

Certainty of the evidence will be assessed through the modified COSMIN Risk of Bias checklist detailed above. Conclusions of methodologically dubious studies will not be considered, while conclusions from methodologically robust studies will be weighted according to the strength of the evidence presented, as determined by an informed qualitative evaluation based on (a) the modified COSMIN Risk of Bias checklist score, (b) the strength of the research designs, and (c) the effect sizes reported in the reliability and validity analyses. This qualitative evaluation of methodological robustness will be summarized in a 4‐point Likert scale with the following labels: (0) methodologically problematic (results should not be considered); (1) methodologically passable (results should be considered as preliminary/exploratory); (2) methodologically adequate (results should be considered); and (3) methodologically robust (results should be given priority). Assessment of certainty of the evidence will be rated by research assistants under the supervision of the co‐lead researcher.

Because methodological concerns can be hard to fully grasp by deciders not familiar with research methods and statistics, the current systematic review will clearly state when, according to strong evidence, a violent radicalization risk tool is fit for purpose and use with a specific population in a specific context. Should no tool have the empirical validation necessary for clear recommendations, the most promising ones according to the evidence will nevertheless be highlighted so that organizations may test drive or use them as part of their multidisciplinary and multidimensional evaluation protocol.

#### 3.6.8 Summary of findings’ tables and evidence profiles

Summary of evidence tables of the retained studies will be prepared for the reader. The first table will present the characteristics and risk of bias of studies, with a clear indication of which ones were discarded after certainty of evidence evaluation. Those that remain will be detailed in the second summary of evidence table, which will list the methodological characteristics of studies (data collection summary and sample description), results classified by reliability and validity types (cf. outcomes), recommendations of authors, and significant limitations.

As in the coding sheets, studies will be aggregated by risk tool. Should a study validate two different risk tools, it will be listed twice (once for each risk tool).

Although not presented in the summary of evidence tables, study limitations not mentioned by the authors but found by the research team will be dealt with thoroughly in the Discussion section. In this way, we will generate ideas that may help improve research designs in the field.

#### 3.6.9 Review author reflexivity (if relevant)

A statement concerning author reflexivity will be included at the beginning of the Methods section if judged relevant by the authors and the Campbell Collaboration's reviewers. There appear to be no conflicts of interest since none of the authors have stakes in or are related to any of the risk tools found in our preliminary searches. Concerning pre‐established positions, our team primarily comprises psychologists and criminologists, most of whom are preoccupied by a “do no harm” approach, which involves staying away from potentially stigmatizing and oppressive approaches. Therefore, concerning risk tools, our recommendations may be more prudent than most. The co‐lead author has substantial expertise in risk assessment in the field of psychology and criminology and this may shape his evaluation of risk tools in the field of violent radicalization. Nevertheless, with the substantial expertise in research methodology shared by multiple members of our team, we are hopeful to avoid most reflexivity pitfalls and assess each study according to criteria as objective as possible.

## CONTRIBUTIONS OF AUTHORS

**Ghayda Hassan (PhD)** will serve as the lead content and systematic review methods expert. Dr. Hassan is a clinical psychologist and professor of clinical psychology at the Université du Québec à Montréal. She is also a researcher, clinician, and policy consultant in matters of interventions in the context of violence (radicalization, family violence, and war), and she has a number of research, clinical, and community‐based national and international affiliations. She is the director of CPN‐PREV (funded by Public Safety Canada). Dr. Hassan is currently serving as a UNESCO co‐chair on Prevention of Radicalisation and Violent Extremism, as well as a researcher and clinical consultant at the SHERPA‐RAPS team and the CIUSSS‐CODIM.

**Sébastien Brouillette‐Alarie (PhD)** will serve as the co‐lead content, systematic review methods, and statistical analysis expert. He is the scientific coordinator of CPN‐PREV and a lecturer at the University of Montréal. His areas of expertise are (a) quantitative research and psychometry, (b) risk assessment of criminal recidivism, (c) prevention of sexual violence, and (d) prevention of violent radicalization. He has also worked as a clinician for the sex evaluation laboratory of the Institut national de psychiatrie légale Philippe‐Pinel. His current research interests include the effectiveness of prevention programs that aim to counter violent radicalization, risk assessment tools for sexual offenders, and criminal desistance.

**Sarah Ousman (MSc)** will serve as a content, systematic review methods, and statistical analysis expert. Sarah is a doctoral student in psychology at UQÀM and an FRQSC scholarship holder. Her thesis, which focuses on the assessment of the risk of violence related to radicalization and extremism, is directed by Ghayda Hassan (UQÀM) and Anne Crocker (Institut national de psychiatrie légale Philippe‐Pinel de Montréal). In addition to her interest in clinical intervention in the context of radicalization and social polarizations, Sarah is interested in transcultural psychology and working with refugee women and marginalized communities.

**Pablo Madriaza (PhD)** will serve as a content, systematic review methods, and statistical analysis expert. Pablo Madriaza holds a bachelor's degree in psychology from the Pontifical Catholic University of Chile, a master's degree in anthropology from the University of Chile, a master's degree in sociology from the École des hautes études en sciences sociales (France), and a PhD in sociology from the University of Paris‐Descartes (France). He has participated in numerous research projects and publications on school violence, crime, and the prison system, especially in an urban context. Recently, he has conducted various studies on preventing radicalization and violent extremism, social conflict, and collective action, as well as the prevention of crime. He has taught in several universities and has participated in several intervention projects principally linked to school violence, domestic violence, and mental health intervention community programs.

**Wynnpaul Varela (PhD)** will serve as a scientific writing expert and research assistant. Wynnpaul Varela is a Montreal‐based independent researcher. His research interests include age‐related differences in musical self‐regulation, systematic reviews, and educational technology. He holds a bachelor's degree in music and a master's in TEFL/TESOL from the University of Birmingham, England, as well as a PhD in education from the University of Concordia, Montréal. He has over 20 years’ language teaching and editing experience in England, France, Japan, and Canada.

**Emmanuel Danis (BSc)** will serve as a research assistant. Emmanuel Danis recently obtained a bachelor's degree in psychology from UQÀM. He joined the CPN‐PREV team to combine his personal interest in violent radicalization with his skills in writing and analyzing scientific literature. His future goal is to pursue a doctorate in clinical psychology, with a focus on cognitive‐behavioral therapy.

**Deniz Kilinc (MSc)** will serve as a research assistant. Deniz Kilinc has completed his master's degree in social and transcultural psychiatry at McGill University. His thesis explored processes of resilience and creative expression on the mental well‐being of Syrian refugee children living outside of refugee camps in Turkey. He is currently completing a law degree at Queens University.

**Eugene Borokhovski (PhD)** will serve as a systematic review methods, statistical analysis, and information retrieval expert. He is an affiliate associate professor at the department of education, Concordia University, and the systematic reviews project manager at the CSLP. He has over 25 years of research experience in social sciences and education, specializing in conducting large‐scale meta‐analyses and mixed‐method systematic reviews in the fields of distance, online, and blended learning, technology integration in education, critical thinking, early literacy, comparative effectiveness of instructional strategies, educational, cognitive, and social psychology.

**David Pickup (MA)** will serve as an information retrieval expert. David Pickup is a librarian working as the information specialist for the systematic reviews team at Concordia University's CSLP, where he also serves a research assistant and maintains the READS collection of ebooks for children (part of the learning toolkit developed by the CSLP). He is also a part‐time faculty member in the department of education, where he teaches an information literacy course. He previously consulted regularly with teams working on Campbell Collaboration systematic reviews, first as the trials search advisor for the Education Coordinating Group, and then with the Disability Group. His research interests include information literacy, systematic review search methodology, and online education.

## DECLARATIONS OF INTEREST

The research team has no potential conflicts of interest for this review. The views expressed are those of the authors and not necessarily of Public Safety Canada or the Campbell Collaboration.

## PRELIMINARY TIMEFRAME

- Draft review submission: December 2022
- Anticipated review publication: May 2023

## PLANS FOR UPDATING THE REVIEW

Ghayda Hassan and her research team will be responsible for updating this review every two years after the initial publication date.

## SOURCES OF SUPPORT

The research team has received funding from Public Safety Canada and approval to move forward with the review.

## Supporting information

Supplementary information.

## Footnotes

1 For more details on the risk‐need‐responsivity principles, consult Andrews and Bonta (2010) or Brouillette‐Alarie and Lussier (2018).
