# Supplementary material for: PROTOCOL: Are tools that assess risk of violent radicalization fit for purpose? A systematic review
Source: Campbell Syst Rev. 2022 Oct 7;18(4):e1279. doi: 10.1002/cl2.1279 (PMC9538709; doi:10.1002/cl2.1279)
Supplement: Supplementary file 1 — Supplementary information. [file CL2-18-e1279-s001.pdf]

Modified COSMIN Risk of Bias Checklist

Violent Radicalization Risk Tools Systematic Review

Items	1 point		0 point		N/A
Risk tool presentation					
1) Is a clear description provided of the construct assessed by the tool?	Yes		No		N/A
2) Is a clear description provided of the target population for which the tool was developed?	Yes		No		N/A
3) Is a clear description provided of the tool's context of use?	Yes		No		N/A
Data analysis (general)					
4) Was an appropriate approach used to analyze the data?	A widely recognized or well justified approach was used	Assumable that the approach was appropriate but not clearly described	Not clear what approach was used or doubtful whether the approach was appropriate	Approach not appropriate	N/A
5) Was the sample size appropriate?	≥ 100	50-99	30-49	<30	N/A
6) Were there any important flaws in the design or statistical methods of the study?	No important methodological flaws		Minor methodolo-gical flaws	Important methodolo-gical flaws	
If there were inter-rater reliability analyses					
7) For dichotomous/nominal/ordinal scores: Was kappa calculated?*	Kappa calculated		No kappa calculated		N/A
8) For ordinal scores: Was a weighted kappa calculated?*	Weighted Kappa calculated		Unweighted Kappa calculated or not described		N/A
If there were internal consistency analyses					
9) Was an internal consistency statistic calculated for each unidimensional scale or subscale separately?	Internal consistency calculated for each unidimensional scale or subscale		Unclear whether scale or subscale is unidimen-sional	Internal consistency not calculated for each unidimen-sional scale or subscale	N/A
10) For continuous scores: Was Cronbach's alpha or omega calculated?*	Cronbach's alpha or omega calculated		Only item-total correlations calculated	No Cronbach's alpha/ omega and no item-total correlations calculated	N/A

11) For dichotomous scores: Was Cronbach's alpha or KR-20 calculated?*	Cronbach's alpha or KR-20 calculated		Only item-total correlations calculated	No Cronbach's alpha/KR-20 and no item-total correlations calculated	N/A
If there were face/content validity analyses					
12) Was each item tested in an appropriate number of participants?	≥ 50	≥ 30	< 30 or not clear		N/A
13) Was an appropriate method used to ask participants about the relevance of each item?	Widely recognized or well justified method used	Assumable that the method was appropriate but not clearly described	Not clear if participants were asked whether each item is relevant or doubtful whether the method was appropriate	Method used not appropriate or participants not asked about the relevance of all items	N/A
14) Was an appropriate method used to ask participants about the tool's comprehensiveness?	Widely recognized or well justified method used	Assumable that the method was appropriate but not clearly described	Doubtful whether the method was appropriate	Method used not appropriate	N/A
15) Was an appropriate method used to ask participants about the comprehensivity of the tool's instructions and items?	Widely recognized or well justified method used	Assumable that the method was appropriate but not clearly described	Doubtful whether the method was appropriate or not clear if participants were asked about the comprehen-sivity of the tool's instructions	Method used not appropriate or participants were not asked about the comprehen-sivity of the tool's instructions and items	N/A
If there were convergent validity analyses					
16) Is it clear what the comparator instrument(s) measure(s)?	The constructs measured by comparators are clear		The constructs measured by comparators are not clear		N/A
17) Was the statistical method appropriate for the hypotheses to be tested?	Statistical method was appropriate	Assumable that statistical method was appropriate	Statistical method applied not optimal	Statistical method applied not appropriate	N/A
If there were comparisons between groups (except cross-cultural validity)					
18) Was an adequate description provided of important characteristics of the subgroups?	Adequate description of the important characteris-tics of the subgroups	Adequate description of most of the important characteris-tics of the subgroups	Poor or no description of the important characteristics of the subgroups		N/A

19) Was the statistical method appropriate for the hypotheses to be tested?	Statistical method was appropriate	Assumable that statistical method was appropriate	Statistical method applied not optimal	Statistical method applied not appropriate	N/A
If there were concurrent/predictive validity analyses					
20) For continuous scores: Were correlations, or the area under the receiver operating curve calculated?*	Correlations or AUC calculated		Correlations or AUC not calculated		N/A
21) For dichotomous scores: Were sensitivity and specificity determined?*	Sensitivity and specificity calculated		Sensitivity and specificity not calculated		N/A
If there were factor analyses					
22) Type of factor analysis performed	Confirmatory factor analysis	Exploratory factor analysis	No exploratory or confirmatory factor analysis performed		N/A
23) Was the sample size appropriate for factor analysis?	7 participants per item and ≥ 100	5 participants per item and ≥ 100; OR 6 participants per item but < 100	5 participants per item but < 100	Less than 5 participants per item	N/A
If there were cross-cultural (external) validity analyses					
24) Were the samples similar for relevant characteristics except for the group variable?	Evidence provided that samples were similar for relevant characteristics except group variable	Stated (but no evidence provided) that samples were similar for relevant characteristics except group variable	Unclear whether samples were similar for relevant characteristics except group variable	Samples were not similar for relevant characteristics except group variable	N/A

**If the statistical analysis employed is not covered here but is still valid, circle the left choice.*

Number of valid items	
Number of points	
**Total score (%)**	
